# Detection of tACS Entrainment Critically Depends on Epoch Length

**DOI:** 10.3389/fncel.2022.806556

**Published:** 2022-03-11

**Authors:** Myles Mc Laughlin, Ahmad Khatoun, Boateng Asamoah

**Affiliations:** Exp ORL, Department of Neurosciences, The Leuven Brain Institute, KU Leuven, Leuven, Belgium

**Keywords:** transcranial alternating current stimulation, neural entrainment, neural oscillations, observation window length, detection of entrainment

## Abstract

Neural entrainment is the phase synchronization of a population of neurons to an external rhythmic stimulus such as applied in the context of transcranial alternating current stimulation (tACS). tACS can cause profound effects on human behavior. However, there remain a significant number of studies that find no behavioral effect when tACS is applied to human subjects. To investigate this discrepancy, we applied time sensitive phase lock value (PLV) based analysis to single unit data from the rat motor cortex. The analysis revealed that detection of neural entrainment depends critically on the epoch length within which spiking information is accumulated. Increasing the epoch length allowed for detection of progressively weaker levels of neural entrainment. Based on this single unit analysis, we hypothesized that tACS effects on human behavior would be more easily detected in a behavior paradigm which utilizes longer epoch lengths. We tested this by using tACS to entrain tremor in patients and healthy volunteers. When the behavioral data were analyzed using short duration epochs tremor entrainment effects were not detectable. However, as the epoch length was progressively increased, weak tremor entrainment became detectable. These results suggest that tACS behavioral paradigms that rely on the accumulation of information over long epoch lengths will tend to be successful at detecting behavior effects. However, tACS paradigms that rely on short epoch lengths are less likely to detect effects.

## Significance Statement

tACS is a promising technique by which brain processing can be engaged and altered. A large number of studies have shown its efficacy of a wide range of brain modalities. However, a significant amount of studies have also failed to find effects on behavior. Here, we used a time sensitive analysis which allowed us to investigate the extent to which observation time affects detection of existing tACS effects. We show that longer duration epochs and larger stimulation strengths allow for detection of entrainment both at the neural and the behavioral levels. This new insight into the temporal aspects of tACS entrainment detection will inform design of studies to better detect tACS effects.

## Introduction

Action potential timing is critical for sensory input encoding (Gray et al., 1989; Williams, 2004; Moxon et al., 2008; Nourski and Brugge, 2011; Steinschneider et al., 2013; Funayama et al., 2015) and neural processing (Hebb, 1949; Volgushev et al., 2016). When action potentials fire in rhythmic bursts they cause neural oscillations (Pettersen and Einevoll, 2008). Ongoing oscillations entrain to rhythmic sensory stimuli, such as speech, flickering lights or tactile input (Regan, 1966; Snyder, 1992; Ahissar et al., 2001; Nourski et al., 2009; Ahn et al., 2016; Minamisawa et al., 2017) by shifting their action potential timing. In addition to sensory stimulation, externally applied electric or magnetic stimulation also influences action potential timing (Fröhlich and McCormick, 2010; Mueller et al., 2014; Vöröslakos et al., 2018; Romero et al., 2019). Transcranial alternating current stimulation (tACS) uses low amplitude sinewave currents to cause neural entrainment. tACS was used to manipulate a range of brain modalities such as auditory, visual and motor function (Wach et al., 2013; Kar and Krekelberg, 2014; Riecke et al., 2015, 2018; Miyaguchi et al., 2018; Dowsett et al., 2020) and also perception and cognition (Neuling et al., 2012; Alekseichuk et al., 2016; Kasten et al., 2016; Kasten and Herrmann, 2017). There is thus considerable evidence that tACS influences behavior by causing neural entrainment. Nevertheless, there remain a number of questions as to its efficacy.

tACS is a nascent field, thus aside from the debate about the cause of neural entrainment, critical questions about the effects of neural entrainment remain poorly understood (Makeig et al., 2004; Philiastides et al., 2010; Papo, 2013). A number of questions can propel better understanding of these questions. For example, how are different brain processes affected by tACS amplitude? Are all neurons equally sensitive to tACS? Providing answers to these questions, in the context of tACS, may help resolve another pressing issue in the tACS field: why do some tACS paradigms affect human behavior (Marshall et al., 2006; Brittain et al., 2013; Pahor and Jaušovec, 2014; Neubauer et al., 2017; Miyaguchi et al., 2018), while others find no effect (Veniero et al., 2017; Bland et al., 2018; Clayton et al., 2018; Sheldon and Mathewson, 2018; Wittenberg et al., 2019).

In current work we addressed some of these questions by investigating the detectability of existing tACS effects. To do this we assessed the ability of the phase lock value (PLV) measure to distinguish between a tACS and a non-tACS condition in epoch lengths of varying duration. In the context of EEG some studies have used computational approaches to highlight the importance of data size in entrainment calculation (Cohen, 2008; Penny et al., 2008). In the context of tACS the analysis here showed that neural entrainment detection depends critically on the observation window or epoch length. To show this we applied a sliding window analysis with varying window lengths. As the window was slid through the spike train we calculated PLV in each window thereby creating an ongoing temporal profile of each neuron’s entrainment. We then compared stimulation-OFF periods to stimulation-ON periods. Shorter duration epochs resulted in higher PLV’s; however, this was spurious and allowed no detection of entrainment. Longer duration epochs, however, allowed for detection of progressively weaker levels of entrainment. Based on this, we hypothesized that entrainment effects on behavior, as quantified by PLV, would be more easily detected using paradigms that rely on longer epoch lengths. We tested this by using tACS to entrain tremor. As predicted, long epoch lengths detected entrainment while shorter windows did not. These new insights significantly advance our understanding of detectability of tACS effects and can be practically applied to develop more robust tACS paradigms.

## Materials and Methods

All data collection methods were described in detail in a previous publication (Asamoah et al., 2019). Datasets presented in that publication, supplemented with newly collected data, were subjected to a newly developed temporal dynamics analysis. The temporal dynamics analyses are described in detail here.

### Rat Neurophysiology

#### Surgery and Experiment Setup

Seven male Wistar rats were used (305–594 g, Janvier Labs) as approved by the KU Leuven animal ethics committee (P096/2015). Rats were anesthetized with ketamine and medetomidine-HCl, fixed in a stereotaxic frame and temperature was monitored *via* a metal rectal probe. Motor cortex forelimb area was targeted for recordings. Two bone screws were implanted in the dried skull and served as tACS electrodes.

#### Stimulation and Recording Setup

Electrical stimulation was delivered with an AM 2200 analog current source. It received a voltage waveform from a data acquisition card (NI USB-6216) which was controlled with a custom MATLAB 2014a software. For recording, a 32-channel silicon probe (E32Tri+R-25-S01-L10 NT, Atlas Neuro) connected to an Intan headstage (RHD2132, intan technologies) and Open Ephys system^1^ were used for recording, amplification, filtering and digitization. Data was visualized online with the Open Ephys GUI.

#### Experimental Protocol

The silicon probe was inserted into the motor cortex at depths from 800 to 2,300 μm. The experimental protocol consisted of 3 consecutive minutes of recording. During the second minute, sinewave stimulation matching the endogenous frequency (1–2.5 Hz) was applied through the stimulation electrodes at amplitudes ranging from 0.025 to 0.5 mA. These amplitudes caused current densities ranging from 0.0167 to 0.33 A/m^2^.

#### Spike Sorting

Spike sorting was performed with the Klusta suite that applied a band pass filter of 300–3,000 Hz which removed the low frequency stimulation artifact. After automatic clustering we did manual curation and analyzed only well isolated clusters.

#### Neural Entrainment Quantification

Single-unit spike times were used to calculate cycle histograms, where one cycle represented one complete sinewave cycle. We extracted entrainment level (phase lock value, PLV) as well as angle of entrainment (phase lock angle, PLA) from the cycle histogram using the equation (Asamoah et al., 2019; Krause et al., 2019; Johnson et al., 2020),


(1) PLV=|∑bRbe|i⁢θb

(2) PLA=angle(∑bRbe)i⁢θb

where θ was the center of bin “*b*” and *R*_*b*_ the normalized magnitude of bin “*b*” such that for the PLV calculation *R*_*b*_ was the likelihood that a spike fell in bin “*b*.” In the pre- and post-stimulation periods, a sinewave was assumed that matched, in phase and frequency, the presented sinewave during the stimulation period.

#### Temporal Dynamics Analysis

**FIGURE 1:**
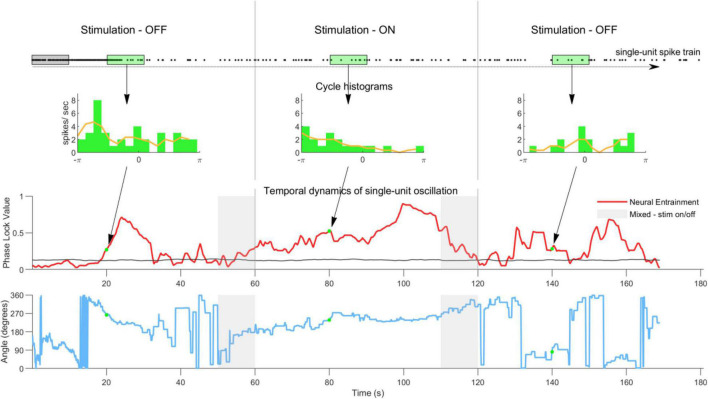
Sliding observation window analysis method used to quantify the temporal dynamics of single-unit neural entrainment. The **first row** shows the single-unit spike-train with each dot representing a spike time. To quantify the temporal dynamics of neural entrainment a 10 s observation window was stepped through the spike train in 10 ms steps. The **second row** shows examples of histograms calculated from the spikes in one observation window (green window). The phase lock value (PLV) and angle were calculated from all histograms. The red line in the **third row** shows the PLV as a function of observation window position. The black line shows the noise floor calculated by randomizing all spike times using a bootstrap method (100 repetitions). The gray areas indicate periods when the observation window contains a mixture of spike times from stimulation ON and OFF periods. These were not taken into consideration for any analysis. Note that in the pre- and post-stimulation periods the PLV is sometimes above and sometimes below the noisefloor, indicating a typical non-stable ongoing neural oscillation. Once stimulation is switched on the PLV quickly increases and remains elevated during stimulation. Once stimulation is switched off the PLV quickly returns to its original level. The **fourth row** shows the phase angle for this example neuron calculated in the same way as the PLV in row three. The angle during stimulation is more stable than in the pre and post conditions. However, it does shift over time (see Supplementary Figure 1 for phase angles at group level).

To elucidate the entrainment temporal dynamics in the spiking activity a sliding observation window analysis was implemented (Figure 1). Within one observation window, spike times were extracted and the PLV calculated using Equation 1. The observation window was then slid 0.01 s forward and the process repeated until the window reached the end of the 3-min recording. This yielded a PLV profile as a function of time (Figure 1, row 3). This analysis was conducted for all single units and all conditions. Due to the sliding nature of the analysis an area contains mixture of data from stimulation-OFF and stimulation-ON periods (see gray areas in Figure 1, third and fourth rows). This area was never taken into account for any analysis. To investigate the effect of the observation window length on neural entrainment, this analysis was repeated using observation windows ranging from 1 to 54 s, in 1 s steps.

#### Neural Population Entrainment Analysis

Using the temporal dynamics profiles, we quantified and ranked the level of neural entrainment within our sample population. To do this, we calculated the mean PLV (PLV_*mean*_), by averaging for each neuron the PLV’s over time, during stimulation and ranked all neurons from high to low PLV_*mean*_. This ranking facilitated division into subpopulations containing response groups of the top 25% or all neurons (100%). Furthermore, the temporal dynamics profiles of all single units were averaged for the subpopulations to get group temporal dynamics for each of the two response groups.

#### Statistical Detection Analysis

Finally, to investigate detectability of tACS neural entrainment, we ran a statistical detection analysis (Harvey, 1992). To do this we calculated a discriminability index (d-prime or d’) between the neural entrainment level in the pre- and during-stimulation periods using the following equation,


(3) $$d^{\prime }=\frac{\mu_{s}-\mu_{n}}{\sqrt{\frac{\sigma \,2_{s}+\sigma \,2_{n}}{2}}}$$

Where μ_*n*_ and σ_*n*_ are, respectively, the mean and standard deviation calculated from the PLV values for the pre-stimulation periods for a given neural population. Similarly, μ_*s*_ and σ_*s*_ are the mean and standard deviation calculated from the PLV values for the during-stimulation periods for the same neural population. We then investigated if the observed levels of neural entrainment could be detected and how the epoch length influences this detection. Finally, we also investigated how neural entrainment detectability is influenced by stimulation amplitude (LOW, MID or HIGH) and the contributing neural population—starting from the most sensitive (as ranked on PLV) 5% of neurons to 100%, in 5% steps.

#### Statistical Analysis of Neural Data

To test whether during the whole stimulation period entrainment was different from non-stimulation periods we applied the Friedman’s test. To distinguish between pre- and during-stimulation periods in the statistical detection analysis for entrainment detection we used a d-prime (d’) measure. d’ = 1 was considered the point of detection.

### Tremor Entrainment Detection in Essential Tremor Patients and Healthy Volunteers

#### Subjects

We recorded pathological tremor from six right handed essential tremor (ET) patients (5 male, 71.8 SD 9.5 years). All patients had DBS electrodes in the ventral intermediate nucleus of the thalamus and had DBS on. We recorded physiological tremor from 12 healthy volunteers (3 male, 10 right-handed, 27 SD 4 years). All experiments were approved by the Medical Ethics Committee at UZ/KU Leuven (S57869) and conducted in accordance with relevant guidelines and the Declaration of Helsinki. All subjects provided written informed consent.

#### Tremor Measurements and Data Acquisition

Tremor was always measured on the dominant hand. Essential tremor patients typically had their arm extended in front of them. Healthy volunteers rested their wrist on a table and had a 15 gram weight attached to their middle finger. Tremor was measured using a tri-axial accelerometer (ADXL335, Analog Devices) attached to the middle finger and digitized (4,096 Hz, NI USB-6216). Each axis was bandpass filtered (3–30 Hz) with a second order Butterworth filter. The first component of a principal components analysis on the three axes was extracted.

#### tACS Electrode Placement and Stimulation

For tACS stimulation a 4 × 1 ring electrode montage was used. Electrodes were gel (Signa Gel, Parker Labs) filled cups. They were placed in an EEG cap (EASYCAP GmbH, Germany) to target the motor cortex contralateral to the dominant hand. For right-handed subjects the center was C3 while for left-handed subjects it was C4. The returns were always the 4 immediately surrounding positions. For stimulation a DS5 current source was driven by earlier mentioned data acquisition card which was controlled *via* custom written MATLAB R2014a software (Mathworks, Natwick, MA). A stimulation OFF condition, during which a gel filled beaker was stimulated, served as control.

#### Experimental Protocol

tACS was always a sinewave at a frequency matching the subject’s individual tremor frequency. For healthy volunteers HIGH was defined as the most tolerable amplitude. LOW was defined as half of HIGH. They underwent three 12 min sessions. Each session contained 3-min of each condition: OFF, LOW and HIGH. For essential tremor patients the HIGH amplitude was 2 mA. Each session consisted of following conditions: 1-min OFF-stimulation, 1-min HIGH-stimulation. This was repeated 5 times. Precise stimulation parameters per subject can be found in Supplementary Tables 4, 5 in Asamoah et al. (2019).

#### Tremor Data Analysis

A Hilbert transform was performed on the tACS and tremor signals to extract instantaneous phase. The phase signals were subtracted to give phase difference as a function of time. The phase differences were separated into 30 bins. All phase differences were normalized to the total amount of time samples to yield the phase difference probability. PLV was then calculated using Equation (1).

#### Population and Statistical Analysis of Human Subject Data

Using the temporal dynamics profiles, we quantified tremor entrainment by calculating the mean PLV (PLV_*mean*_). To do this we averaged the temporal PLV’s we calculated for each subject separately. To determine the epoch length necessary to detect an existing effect we applied the following statistics: For the pathological tremor data we used a Wilcoxon sign-rank test to compare OFF and HIGH PLVs. For the physiological tremor data we applied a linear mixed model to compare OFF, LOW and HIGH PLVs. For all tests α = 0.05.

#### Vector Sum PLV (PLV_*VecSum*_) to Detect Differences in Entrainment

When assessing the effect of epoch length on entrainment detection we calculated the average PLV over all epochs without taking into account the phase angle of each individual epoch, i.e., we calculated the magnitude of the complex value for each individual epoch (see Equation 1, defined simply as PLV) and took the average of the magnitudes. This standard PLV approach does not take into account the individual epoch phase angle of entrainment (see Equation 2), which could differ from one individual epoch to the next (see Figure 1). Therefore, in a follow-up second approach we calculated the average PLV by first computing a vector sum of the complex value (containing both magnitude and angle) and then dividing by the total number of epochs. We call this the vector sum PLV (PLV_*VecSum*_). The second approach, using PLV_*VecSum*_, thus investigates whether the effect of angle variation between each epoch has an effect on the entrainment detection.

## Results

### Entrainment Effects Depend on Stimulation Amplitude

**FIGURE 2:**
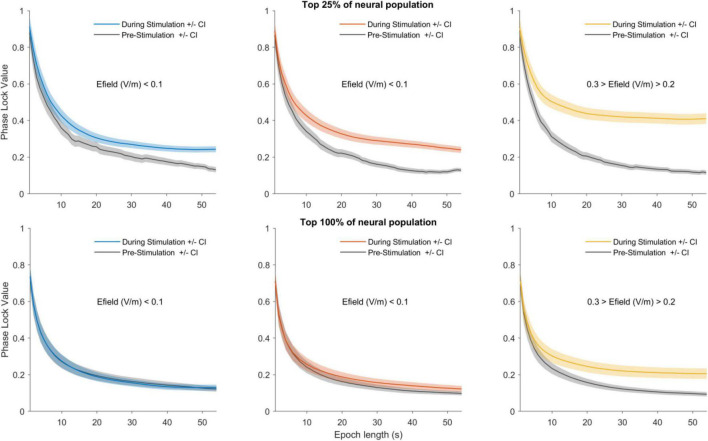
Detection of neural entrainment depends on epoch length and stimulation strength. Upper row shows the 25% most sensitive neurons and the lower row shows the 100% most sensitive neurons (all neurons). For an observation window duration (e.g., 1 s) the group temporal dynamics was calculated. The mean PLV for the entire pre- and during-stimulation periods were then calculated. This procedure was then repeated for observation windows ranging from 2 to 54 s. The mean pre-stimulation PLV as a function of observation window duration is shown in gray and the during-stimulation PLV is shown in color: blue for LOW amplitude stimulation (left column), red for MID (middle column) and yellow for HIGH (right column). The shaded areas depict 95% confidence interval. The main trends show short observation windows lead to higher PLVs but only small differences between the pre- and during-stimulation phases. As the observation window duration is increased the PLV levels decrease, but importantly the difference between pre- and during-stimulation becomes larger. Note also that as stimulation amplitude is increased the difference between pre- and during PLVs become larger. Thus, longer observation windows and higher stimulation amplitudes make the difference in pre- and during PLVs larger.

Figure 2 shows group data from neurons in the top 25% (upper row) and top 100% (all neurons) (lower row) of the PLV. The left, middle and right panels show entrainment of data by LOW, MID and HIGH amplitude stimulation levels which corresponded to approximately 1 V/m, between 1 and 2 V/m and between than 2 and 3 V/m (Asamoah et al., 2019). We first ranked all neurons from highest to lowest PLVs in the during condition (see section “Materials and Methods,” Neural Population entrainment analysis). We then performed statistics on the 25% of the neural population showing the highest PLVs by comparing PLVs in the pre-, during- and post-stimulation periods. HIGH stimulation showed a significant effect (Friedman’s test, p < 0.001, χ^2^ = 21.3). Bonferroni corrected *post-hoc* tests showed that during-stimulation group mean PLV was different from pre- and post-stimulation group mean PLV (pre- to during-stimulation *p* < 0.001; during- to post-stimulation *p* < 0.001). Pre- and post-stimulation PLVs, however, did not differ (*p* = 1). A similar effect was detected for MID stimulation (Friedman’s test *p* < 0.001, χ^2^ = 23.6; *post-hoc*: pre- to during-stimulation *p* < 0.001, during- to post-stimulation p < 0.001, pre- to post *p* = 1). The effect was absent for LOW stimulation (Friedman’s test *p* = 0.171, χ^2^ = 3.5). Stimulation did not change spike-rate (HIGH—Friedman’s test *p* = 0.348, χ^2^ = 2.1); (MID—Friedman’s test *p* = 0.410, χ^2^ = 1.8); (LOW—Friedman’s test *p* = 0.827, χ^2^ = 0.38). In other words, as shown earlier sinewave stimulation up to electric field values of 3 V/m can entrain neurons but do not initiate action potentials; rather, spike timing is altered. 100% of the data showed similar trend (Statistics are shown in Table 1).

**TABLE 1:** Friedman and *post-hoc* test for PLV from top 25 and 100% of the neural population.

PLV		25%			100%	
		
	Friedman: *P*-val (χ^2^)					
LOW	0.171 (3.5)			0.395 (1.9)		
MID	<0.001 (23.6)			<0.001 (15.6)		
HIGH	<0.001 (21.3)			<0.001 (31.1)		

	***Post-hoc*: Bonferroni corrected *P*-val**					

	***Pre—Dur***	***Pre—Post***	***Dur—Post***	***Pre—Dur***	***Pre—Post***	***Dur—Post***

LOW	0.269	1	0.369	0.956	0.577	1
MID	<0.001	1	<0.001	0.015	0.949	<0.001
HIGH	<0.001	1	<0.001	<0.001	1	<0.001

### Neural Entrainment Detection Depends on Epoch Length

tACS effects are often difficult to detect. To understand why, we investigated the effect of observation window duration on neural entrainment detection by comparing PLV_*mean*_ in the pre- and during-stimulation periods for different window durations. Figure 2 upper panel, shows the results for the 25% population. This analysis highlights two phenomena: Firstly, the observation window length influences calculated PLV—shorter windows show higher PLVs while longer windows show lower PLV values. This is, however, a necessary consequence of the PLV calculation and no conclusions can be drawn from it (see section “Discussion”). Secondly, and more importantly, the difference between neural entrainment in the pre- and during-stimulation periods becomes larger for longer observation windows and higher stimulation amplitudes. This indicated that longer observation windows are better suited for detecting small neural entrainment effects. The same trends are visible when the entire neural population (100%) was included in the analysis (Figure 2, lower panel). However, the observation window length needed to detect a difference between the pre and during conditions is always longer than for the top 25% of neurons.

**FIGURE 3:**
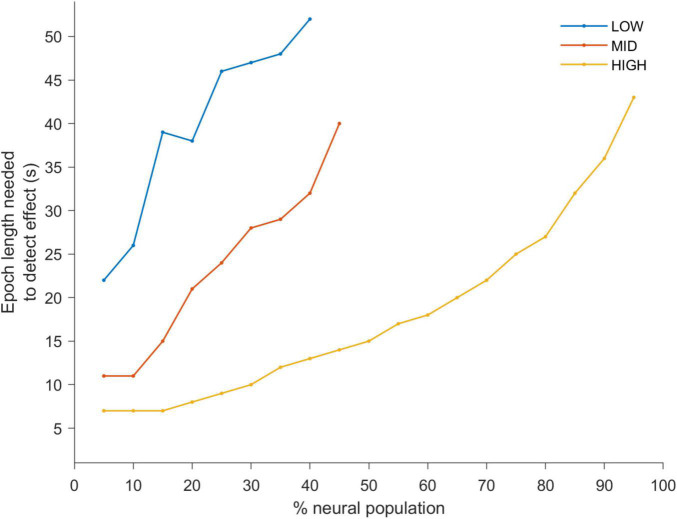
A d-prime analysis was used to determine a statistical detection change in PLV between the pre- and during-stimulation periods. The lines of the plots indicate the shortest observation window duration to reach a d-prime value of 1 (i.e., the point of statistical discrimination between the pre- and during-stimulation PLV’s) as a function of the percentage of the neural population included in the analysis (ranked from highest to lowest PLV). This is shown for LOW (blue), MID (red) and HIGH (yellow) stimulation amplitudes. This quantitative analysis confirms the trends apparent in Figure 2. Namely, that with increasing stimulus amplitude the observation window needed to discriminate between the two conditions becomes smaller. It is also apparent that neurons which are most sensitive to stimulation reach the point of discrimination with short epoch lengths while a larger percentage of the neurons (including less responsive neurons) need a larger epoch lengths to do so.

To quantify this we applied a statistical detection analysis (Figure 3), where d’ = 1 is the detection point for pre- and during-stimulation PLV changes. Thus, each line indicates the minimum observation window duration to reach d’ = 1 as a function of the percentage of the neural population included in the analysis. As the observation window length and stimulation amplitude increase, entrainment detection becomes easier, e.g., at HIGH levels (yellow-line) the most sensitive neurons in the population (5–25%) can detect entrainment changes in less than 10 s. However longer observation windows are needed as the stimulation amplitude is decreased (red- and blue-lines). Furthermore, as more of the less sensitive neurons are included (i.e., moving from left to right along the *x*-axis) longer windows are needed to detect a change in entrainment.

### Tremor Entrainment Detection Depends on Epoch Length

**FIGURE 4:**
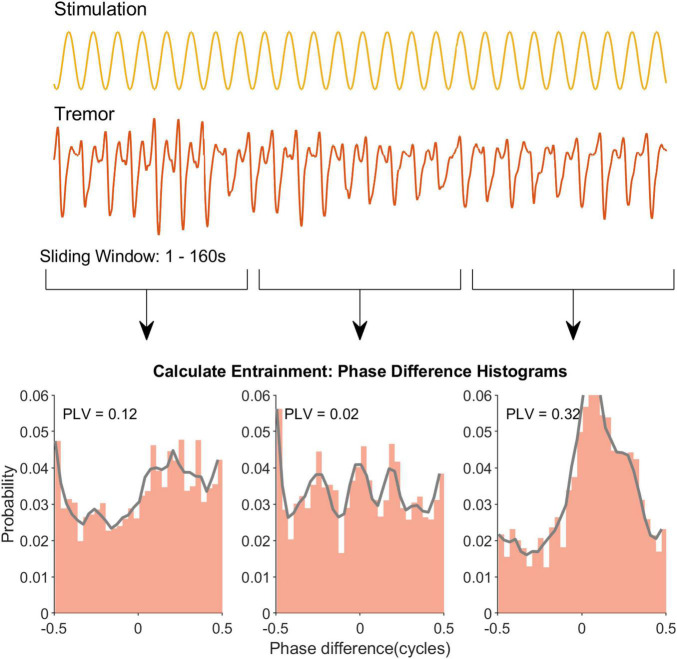
Sliding window analysis to extract the temporal profile of tremor entrainment in an example recording. The first two rows show the sinewave stimulation and the recorded tremor respectively. Note that for clarity only a segment of each signal is depicted. Similar to Figure 1 for the neural data a window slides through the recording and a PLV is calculated for that window (row 3). Eventually this yields a temporal profile for this example recording.

**FIGURE 5:**
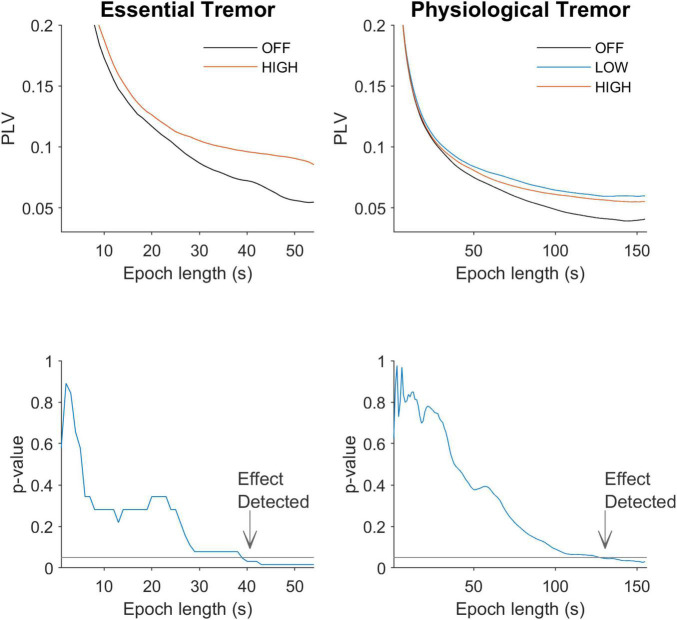
Effect of epoch length on detection of differences between conditions. The **left column** shows data from tACS in essential tremor patients. The **right column** shows data for tACS applied to healthy volunteers. The upper row shows entrainment levels in PLV as a function of epoch length. Similar to the spike train data in Figure 3, shorter epochs give higher PLVs but the difference between OFF and ON is small. As the epoch becomes longer in duration the difference between stimulation OFF and ON becomes larger. The lower row shows the *p*-values associated with the different epoch lengths and quantitatively confirms this observation. The horizontal line at *p*-value of 0.05 shows the alpha level.

Based on the analysis of the neural data we predicted that behavioral paradigms that use (1) long duration epochs and (2) high amplitude stimulation will be best suited to detect entrainment effects. To test this prediction we reanalyzed essential tremor tACS data—which had already shown entrainment (Asamoah et al., 2019)—using a range of different epoch lengths (Figure 4). The average PLV for all patients is shown as a function of epoch length (middle column, upper panel). Similar patterns with the rat neurophysiology data are apparent (compare Figure 2 with Figure 5, upper row). For essential tremor data we applied the Wilcoxon signed rank to test between the stimulation-OFF and stimulation-ON conditions. Figure 5, lower left column shows that entrainment is only detected (*p* ≤ 0.05) when epoch length is around 39 s long. The right column shows physiological tremor data. The upper right panel shows the same trend as in the essential tremor and neural data. To test for statistical detection, here we applied the linear model on the conditions OFF, LOW and HIGH. Similar to the essential tremor data we see that effect detection (*p* ≤ 0.05) only occurs at longer epochs rather than smaller. These results indicate that when data collection time is limited, optimizing tACS behavioral paradigms to have less repetitions but longer epoch durations, will increase the likelihood of detecting an entrainment effect.

### Effect Detection With PLV_*VecSum*_ Also Depends on Epoch Length

To investigate whether, in principle, the PLV_*VecSum*_ approach effects detection of effect differently from the standard PLV approach we applied a vector sum calculation to the same neural data (see section “Materials and Methods,” Vector sum PLV (PLV_*VecSum*_) to detect differences). The patterns shown by this angle sensitive approach are the same as the standard PLV approach such that longer duration epochs allow detection of effects while shorter epochs fail to do so (Supplementary Material).

## Discussion

Here, we used neural spiking and human tremor data to show that detection of tACS entrainment, as quantified through PLV, depends on observation window and epoch length and stimulus amplitude. Increasing observation window and tACS amplitude facilitates detection of weak entrainment.

In the tACS literature neural entrainment, as one static measure over a whole recording condition, is determined by comparing stimulation-OFF to stimulation-ON periods (Asamoah et al., 2019; Johnson et al., 2020; Vieira et al., 2020). This approach is necessary because studies typically find entrainment levels in the stimulation-OFF periods (although there is no entraining signal). This has two distinct causes; (1) Due to chance, it is expected that some entrainment levels are found even when there is none. (2) The entraining signal frequency is typically chosen to be close to the ongoing oscillation frequency in the stimulated brain region. This increases the likelihood that by chance entrainment is found even when there is none. This is the case when static entrainment was calculated over whole recording periods (Ozen et al., 2010; Asamoah et al., 2019; Vieira et al., 2020) but we also found this in our temporal dynamics approach.

When we calculated entrainment using short observation windows PLV tended to be high. As the window was increased PLV gradually became smaller until it stabilized when the window was sufficiently large; thereby giving a distinct PLV trend. This trend occurred when there was no stimulation (pre-condition) but also appeared when there was stimulation (during-condition). This is a necessary consequence of the PLV calculation and as such both the neurophysiological and the behavioral data showed this trend. Intuitively when one spike occurs during the whole recording this results in a PLV of 1. The smaller the amount of spikes the more likely it is that the PLV is high. However, as the amount of spikes increase the calculated PLV drops to a stable level. Notably, further increasing the window length does not cause further decrease of PLV. This shows that once there is sufficient data to calculate a reliable entrainment the PLV only reflects entrainment values in the data and is no longer depended on the observation window length. The window length is important for a reliable PLV calculation because the experimenter, given a defined set of experimental conditions, has no control over spike rate and therefore data size. As such, no conclusions can be drawn on the basis of the PLV trend visible in Figure 2 as such. Rather, attention should be paid to the difference between stimulation-OFF and stimulation-ON periods. As the observation window length increases a clear difference between the two conditions becomes apparent (reflecting there is a real difference in entrainment during stimulation-ON and -OFF periods). This is also the basis of the dependency of detectability on observation window length. When the amount of data (spikes in Figure 2) is insufficient for a stable PLV calculation there is no difference between the manipulation and the control conditions. We can therefore only draw conclusions by contrasting stimulation-OFF conditions with stimulation-ON conditions.

In the context of EEG data computational approaches showed earlier that detection of entrainment can depend on data length and therefore observation window length (Penny et al., 2008). Here we used neural and human tremor data to show that this is also the case in tACS entrainment. We showed that detection of tACS effects is an interplay between observation window and epoch length as well as stimulation amplitude.

Many tACS studies have highlighted the potential of the technique to modulate a range of behavioral functions (Wach et al., 2013; Kar and Krekelberg, 2014; Alekseichuk et al., 2016; Miyaguchi et al., 2018; Riecke et al., 2018), while many others fail to find any effect (Veniero et al., 2017; Bland et al., 2018; Clayton et al., 2018; Fekete et al., 2018; Sheldon and Mathewson, 2018; van Schouwenburg et al., 2018; Wittenberg et al., 2019). Thus, to advance the field it is essential to both understand the mechanism through which tACS causes neural entrainment (Khatoun et al., 2019; Krause et al., 2019) as well as the factors which influence entrainment detection. Typically tACS neurophysiological studies use observation window lengths that match in duration the entire stimulation period (Fröhlich and McCormick, 2010; Ozen et al., 2010; Asamoah et al., 2019; Krause et al., 2019). These approaches are relevant for brain processes that take place over longer timescales, such as learning and reasoning (Makeig et al., 2004; Papo, 2013), but miss changes in neural entrainment that are important for brain processes occurring over shorter periods of time; such as perception and detection (Cohen, 2011; Kelly and O’Connell, 2015). We developed a temporal dynamics analysis that is sensitive to fast changes in entrainment and may be more relevant for understanding tACS effects on perception and detection. Specifically, we showed that for statistical detection of tACS induced changes in neural activity we would need long time periods (10’s of seconds) to detect entrainment when stimulation intensities are low. However, as the stimulation intensity increases shorter observation windows are sufficient to detect changes in the neural activity (Figure 3).

Our rat neurophysiological experiments highlighted the importance of epoch length for tACS neural entrainment using PLV. In the healthy volunteer and patient tremor experiments we showed that epoch length is also critical for detecting tACS effects on human behavior. When detection time is short experiments may be repeated to allow detection of existing effects. Our data and analysis here show that the low amplitude nature of tACS allows better detection with longer observation windows rather than shorter; even when total amount of data does not change. The dependency of the PLV metric on epoch length can likely be generalized to other research paradigms where this metric is applied. The optimal epoch length will likely depend on specific experimental conditions and should be taken into consideration in pilot studies. For tACS studies that do not use this metric it is not necessarily clear that epoch length determines detectability.

## Data Availability Statement

The raw data supporting the conclusions of this article will be made available by the authors, without undue reservation.

## Ethics Statement

The studies involving human participants were reviewed and approved by the Medical Ethics Committee at UZ/KU Leuven. The patients/participants provided their written informed consent to participate in this study. The animal study was reviewed and approved by KU Leuven animal ethics committee.

## Author Contributions

All authors listed have made a substantial, direct, and intellectual contribution to the work, and approved it for publication.

## Conflict of Interest

The authors declare that the research was conducted in the absence of any commercial or financial relationships that could be construed as a potential conflict of interest.

## Publisher’s Note

All claims expressed in this article are solely those of the authors and do not necessarily represent those of their affiliated organizations, or those of the publisher, the editors and the reviewers. Any product that may be evaluated in this article, or claim that may be made by its manufacturer, is not guaranteed or endorsed by the publisher.

## Footnotes

1
http://www.open-ephys.org/store
