# Supplementary material for: Detection of tACS Entrainment Critically Depends on Epoch Length
Source: Front Cell Neurosci. 2022 Mar 11;16:806556. doi: 10.3389/fncel.2022.806556 (PMC8963722; doi:10.3389/fncel.2022.806556)
Supplement: Supplementary file 1 [file Data_Sheet_1.docx]

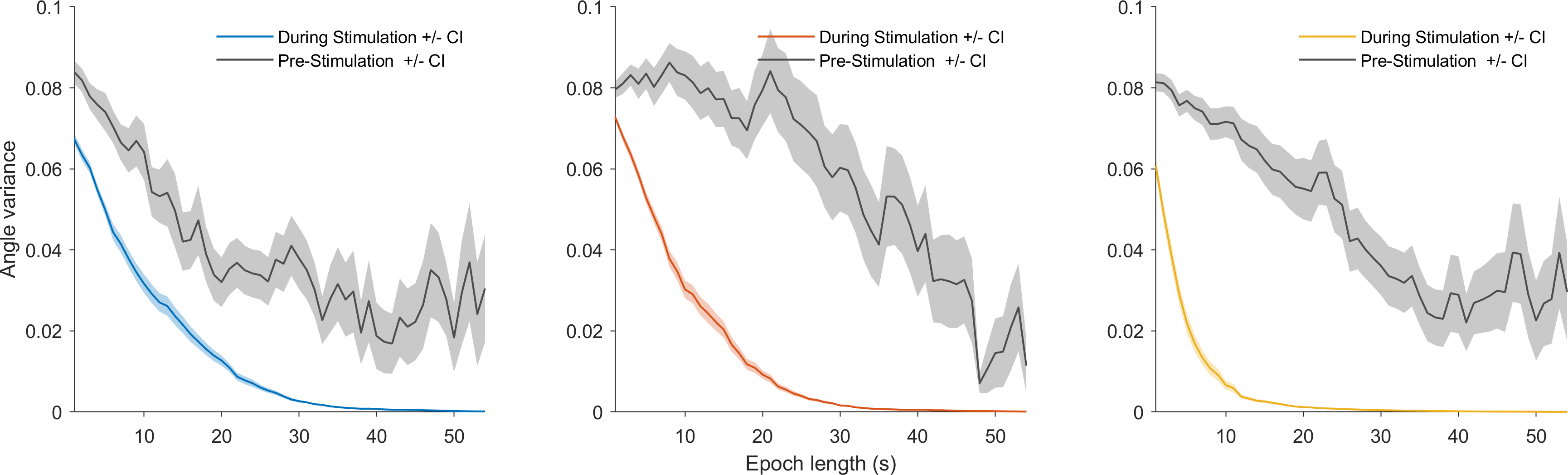


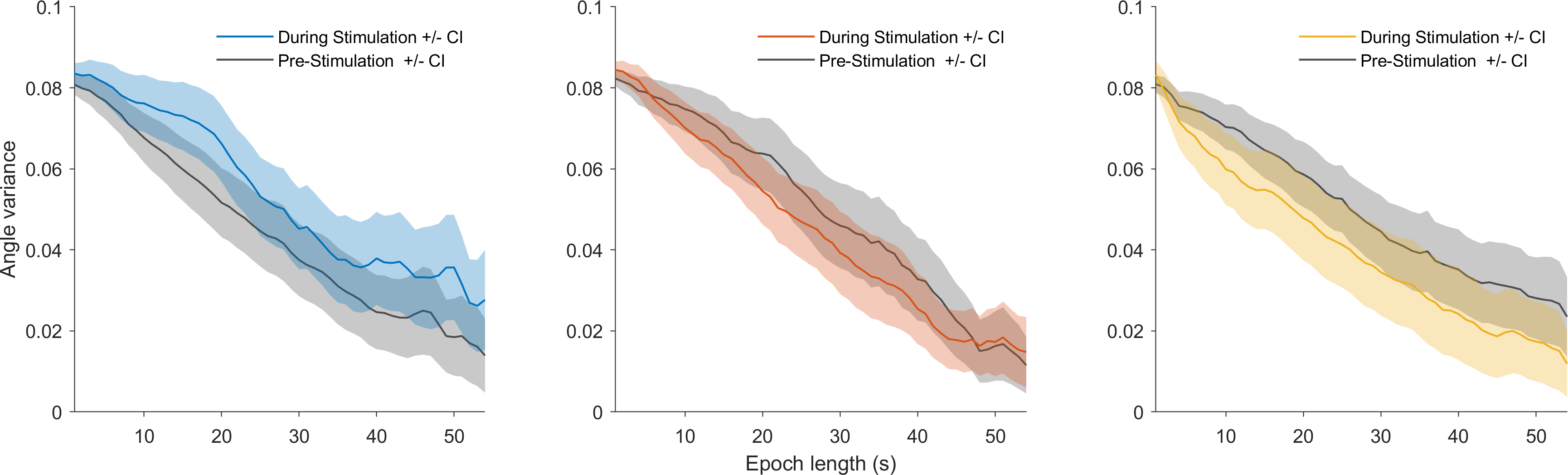


Supplementary Figure 1. Stability of entrainment angle during stimulation. In all graphs the y-axis shows the variance of the entrainment angle as a function of epoch length. We calculated the variance of the angle for all recordings separately and then ranked neurons from low to high variance. A high variance indicates that the angle of entrainment changed considerably (little angle stability) during the recording while a low variance implies that there was less change in the angle of entrainment (high stability). The **upper row** of the graph shows the top 25% of neurons with the most stable angle while the **lower row** shows the variance of all neurons. The blue, red and yellow depict stimulation intensities of LOW, MEDIUM and HIGH. Firstly, as the epoch length increases angle variance decrease; which means that angle stability increases. To determine whether stimulation impacted angle stability we therefore compare the stimulation conditions (blue, red, yellow) with the non-stimulation conditions in grey. In the upper row there is a difference between the stimulation and non-stimulation conditions. This implies that in this group of neurons the entrainment angle stabilizes. In the lower row, however, there is no difference between the two conditions (per graph). This implies that when all neurons are taken into consideration entrainment angle does not stabilize. This figure shows that although the angle of some neurons may be stable (the pre- and during-stimulation conditions differ), overall the angle of entrainment does not stabilize (in the lower row there is no difference between the two conditions).


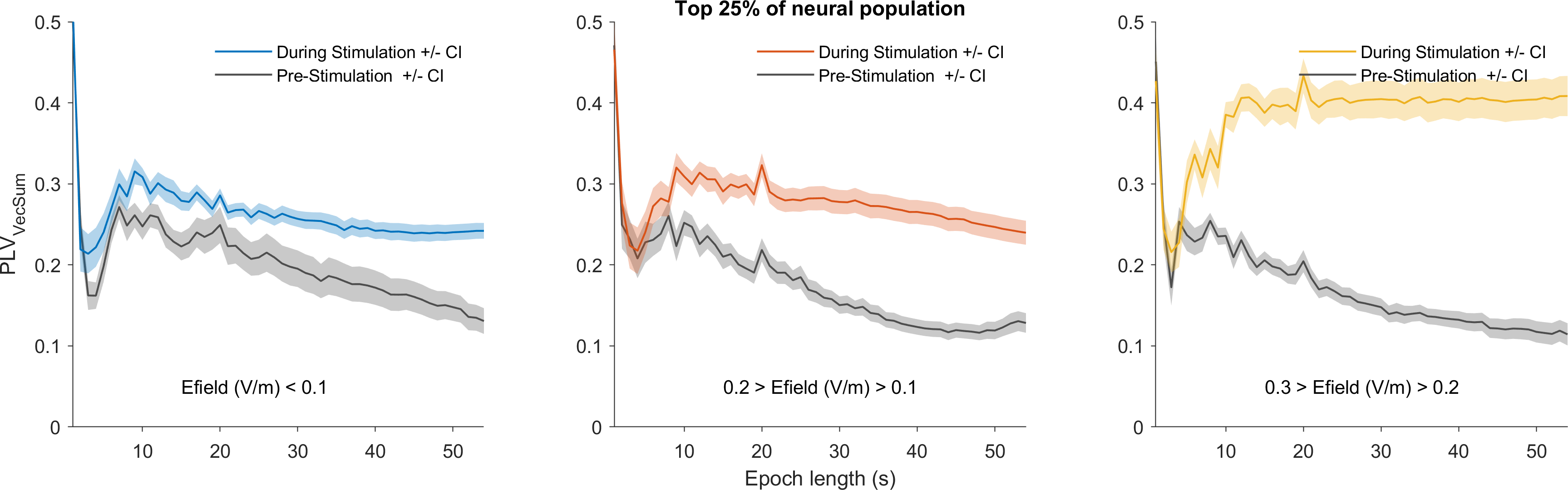


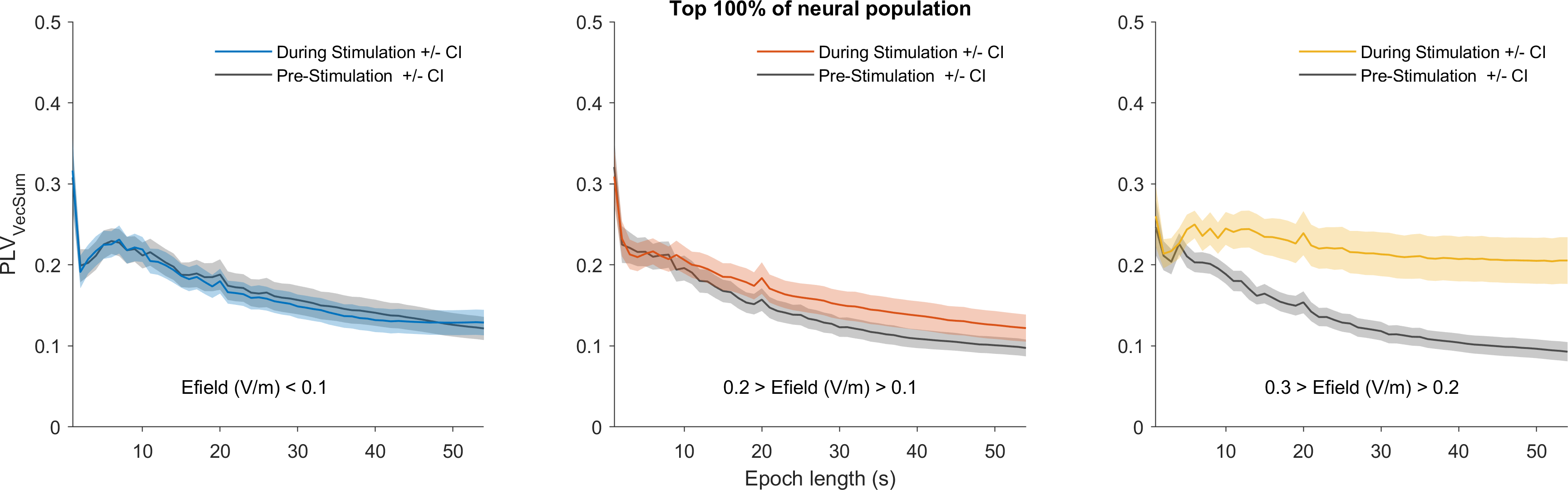


Supplementary Figure 2 Detection of neural entrainment (as calculated using the vectorized sum approach (see in the main text Methods, Vector sum PLV (PLV_VecSum_) to detect differences in entrainment)) depends on epoch length and stimulation strength. This figure is structured in the same way as Figure 2 in the main manuscript. Note that while the initial PLV_VecSum_ PLV’s for short observation windows are lower than in Fig 2 in the main manuscript (PLV), the general patterns are similar – namely the difference between pre and during conditions increase with longer observation windows and higher stimulation amplitudes. This thus result in the same conclusion; larger epoch lengths and higher stimulation amplitudes allow better detection of differences.


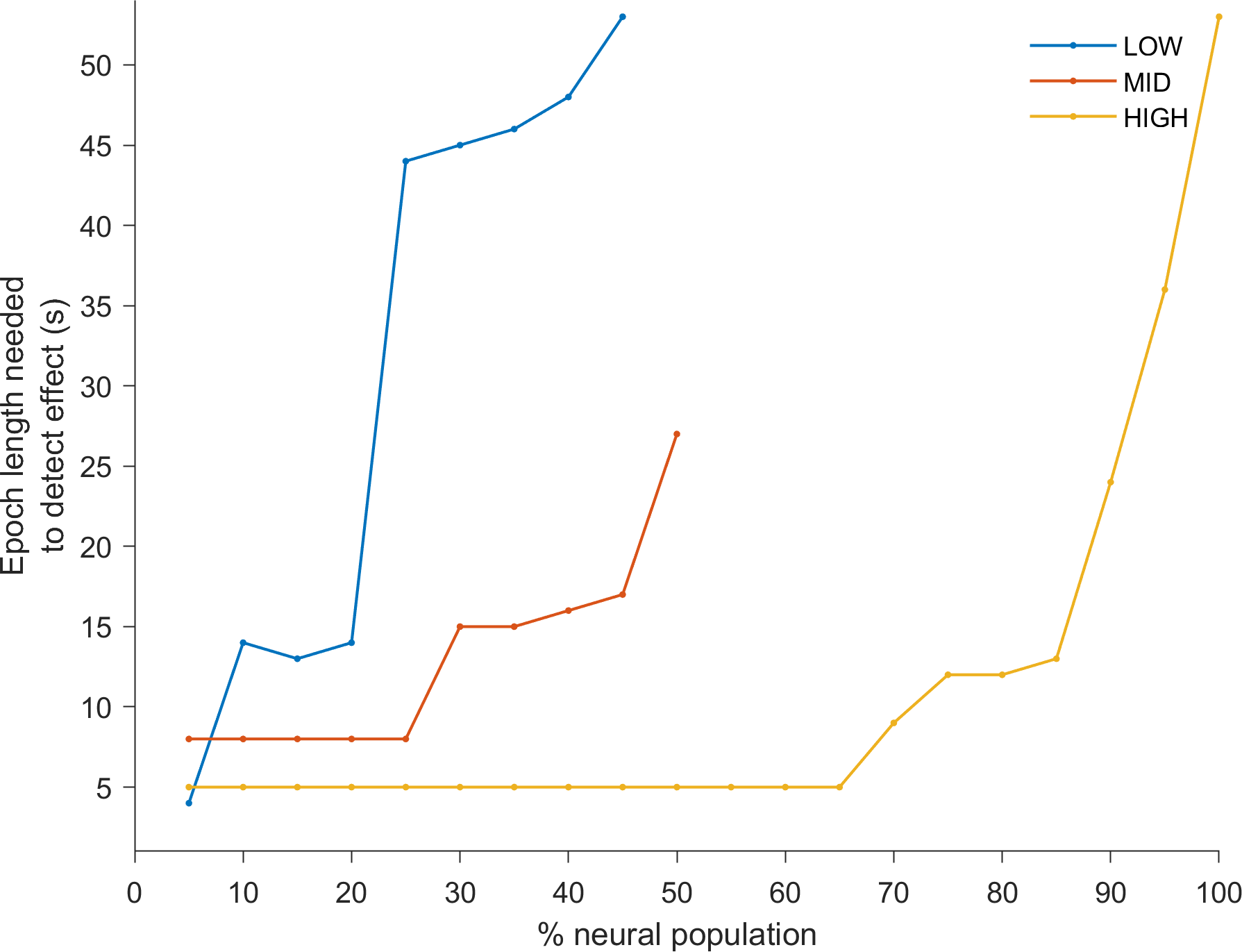


Supplementary Figure 3. d-prime analysis to determine a statistical detection change in PLV_VecSum_ between the pre- and during-stimulation periods. This figure is structured in the same way as Figure 3 in the main manuscript and also shows the same patterns. With increasing stimulus amplitude the observation window needed to discriminate between the two conditions becomes smaller. It is also apparent that neurons which are most sensitive to stimulation reach the point of discrimination with short epoch lengths while a larger percentage of neurons (including less responsive neurons) need larger epoch lengths to do so.
